# Supplementary material for: Granulovirus PK-1 kinase activity relies on a side-to-side dimerization mode centered on the regulatory αC helix
Source: Nat Commun. 2021 Feb 12;12:1002. doi: 10.1038/s41467-021-21191-7 (PMC7881018; doi:10.1038/s41467-021-21191-7)
Supplement: Supplementary file 1 — Supplementary Information [file 41467_2021_21191_MOESM1_ESM.pdf]

SUPPLEMENTARY FIGURES AND LEGENDS, TABLES AND REFERENCES

SUPPLEMENTARY FIGURES AND LEGENDS

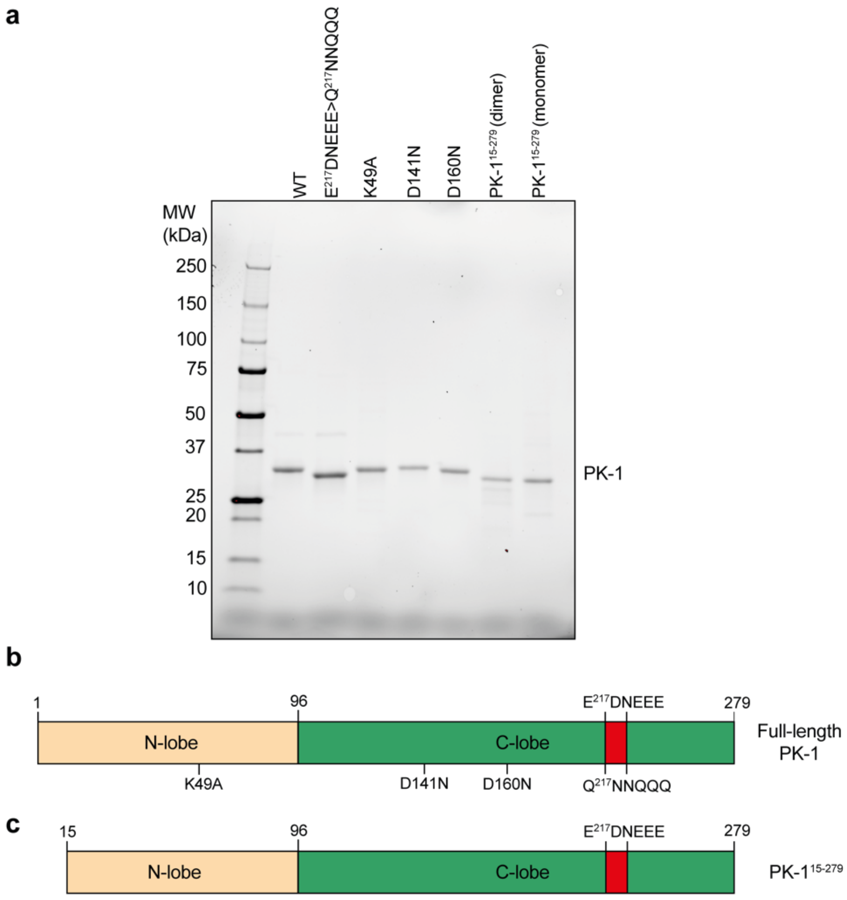

Supplementary Figure 1 | Purification of wild-type PK-1 and mutant constructs. a) Reducing SDS-PAGE analysis of the purified PK-1 constructs visualized by stain-free imaging. Positions of molecular weight standards (first lane) are annotated on the left of the gel. The purity of the shown protein preparations are representative of at least 3 independent expressions and purifications for each construct. **b)** Domain organisation highlighting all single-point mutations in the N-lobe (beige) and C-lobe (green). The acidic patch is highlighted in red. Numbers and amino acids above the domain organisation are associated with the wild-type PK-1 sequence, while the mutations are shown below. **c)** PK-1$^{15\text{-}279}$ construct in which the N-terminal helix has been truncated.

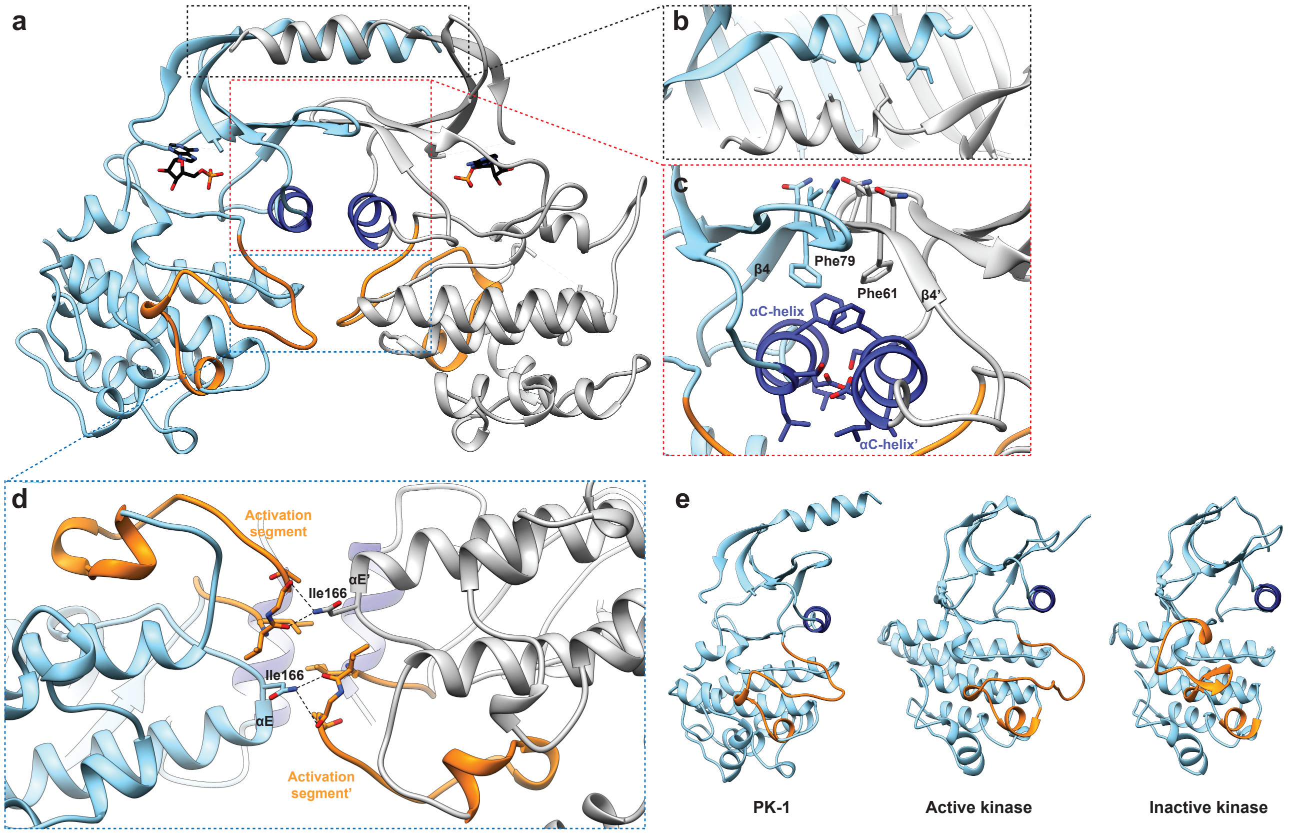

Supplementary Figure 2 | The PK-1 dimeric interface. a) Cartoon representation of the PK-1 dimer (blue and grey) and the functionally important αC helices (blue) and activation segment (orange). The key regions of the dimeric interface are shown in **b-d)**, which include: a domain-switched interface formed by an exchange of the N-terminal α-helices between monomers (**b**); both an extension of β4-sheet and a phenylalanine-rich hydrophobic core formed between β4-sheet and the αC-helix (**c**); and interaction between the activation segment of each monomer, largely mediated by Ile166 (**d**). **e)** Comparisons of PK-1 and active (PDB ID: 2F4J)[1] or inactive (PDB ID: 2HYY)[2] conformers of Abl kinase demonstrate that PK-1 adopts an open, active activation loop conformation, rather than a closed, inactive conformation.

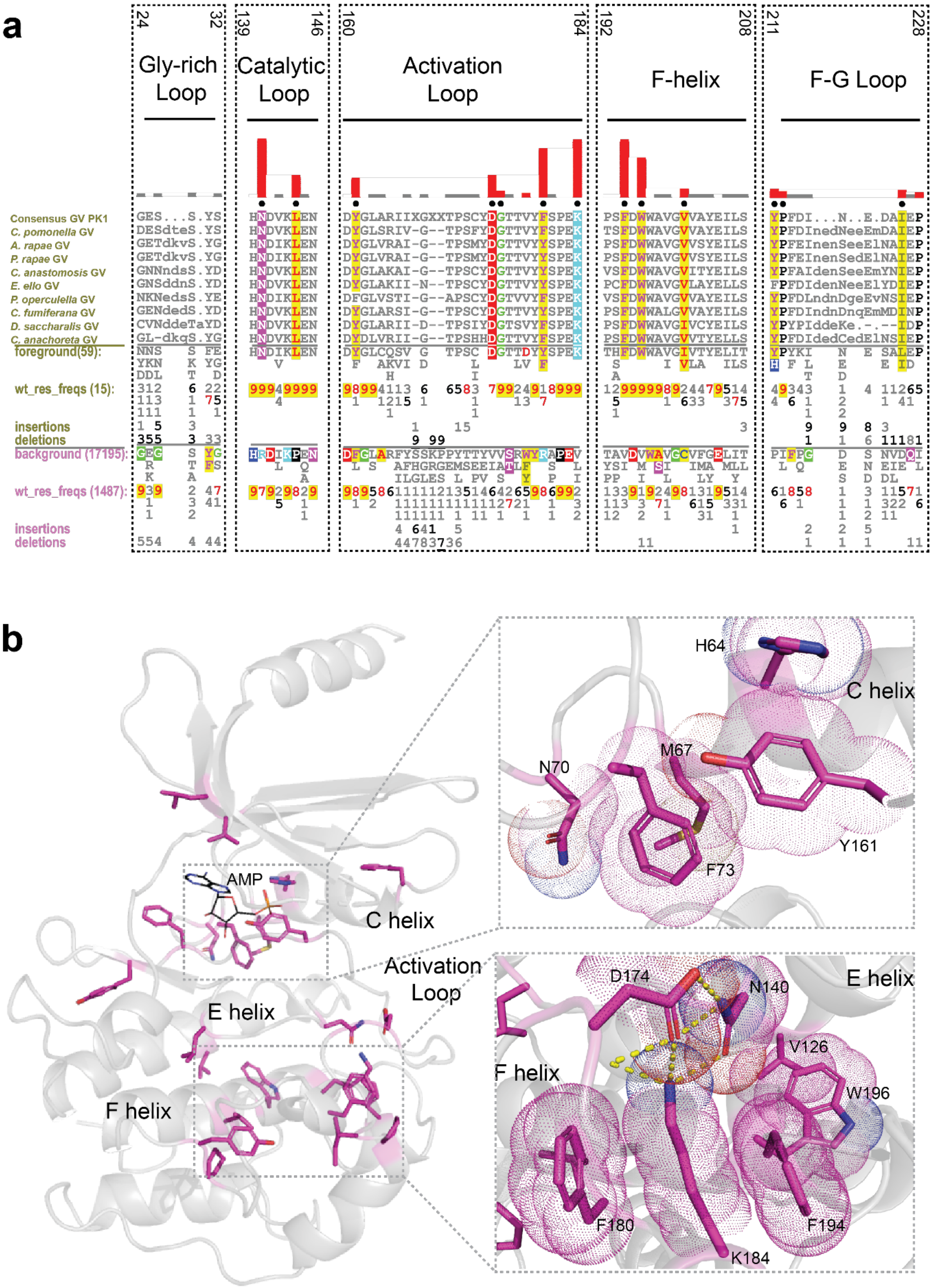

Supplementary Figure 3 | PK-1 specific sequence constraints potentially stabilize the activation loop in the extended state. **a)** Sequence constraints that distinguish *Cp*GV PK-1 from other baculovirus PK-1 sequences are shown as a contrast hierarchical alignment (CHA)[3]. The CHA shows

selected PK-1 sequences from various baculovirus species as the display alignment, a foreground alignment of 59 PK-1 sequences and a background alignment of 17195 sequences. The foreground and background alignments are shown as residue frequencies below the display alignment in integer tenths (1–9). The histogram (in red) above the display alignment indicates the extent to which the distinguishing residues in the foreground alignment diverge from the corresponding position in the background alignment. Black dots mark the alignment positions used by the BPPS procedure[4] when classifying PK-1 from other PK-1 sequences. Alignment numbering (top) is based on the *Cp*GV PK-1 sequence. Accession numbers for the sequences are as follows: *A. rapae* PK-1: YP_003429327.1; *P. rapae* PK-1: AGS18769.1; *C. anastomosis* PK-1: YP_009505945.1; *E. ello* PK-1: ARX71862.1; *P. operculella* PK-1: NP_663168.1; *C. fumiferana* PK-1: YP_654424.1; *D. saccharalis* PK-1: YP_009182201.1; *C. anachoreta* PK-1: YP_004376211.1. **b)** Cartoon representation of chain A of *Cp*GV PK-1 dimer. Sequence constraints were mapped onto the structure with PK-1 specific residues shown as sticks and colored in magenta. (Insets) Zoomed in view of the two separate clusters of interaction N- (top) and C- (bottom) termini of the activation loop. The residues are further shown as dots to visualize van der Waals interactions. Hydrogen bonds are shown with dashed yellow lines.

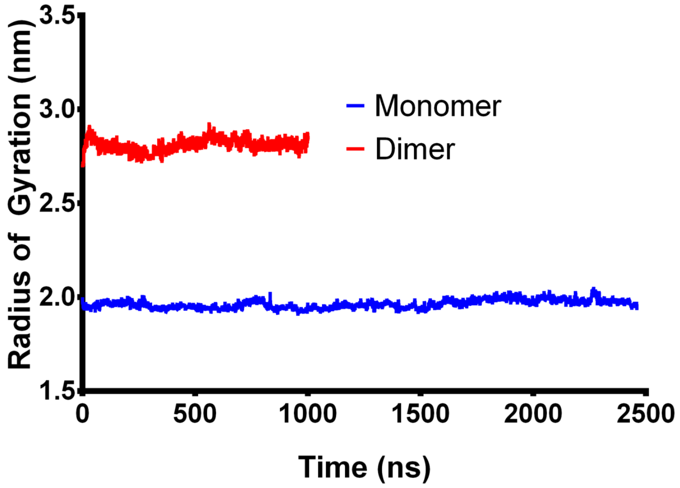

Supplementary Figure 4 | The radius of gyration (Rg) for monomeric PK-1 (blue) and dimeric PK1 (red) in MD simulations.

SUPPLEMENTARY TABLES

Supplementary Table 1 | Oligonucleotide and optimized PK-1 template sequences

PK-1 fwd (Gateway)	5´-ggcagcggcgcgatgaatcccagtaaatcc-3´
PK-1 rev (Gateway)	5´-gaaagctgggtgttaaaaacataaaaatgggtg-3´
PK-1 BamHI 2 fwd‡	5´-CGCGGATCCaatcccagtaaatccatttcgcg-3´
PK-1 279 stop EcoRI rev	5´-CGCGAATTCAaaaacataaaaatgggtgtttaattatttc-3´
PK-1 D141N fwd	5´-caatattgtgcacaacAacgtcaaactggaaaac-3´
PK-1 D141N rev	5´-gttttccagtttgacgtTgttgtgcacaatattg-3´
PK-1 D160N fwd	5´-gactgtttgtatgcAactacggactgtcg-3´
PK-1 D160N rev	5´-cgacagtccgtagtTgcatacaaacagtc-3´
PK-1 codon optimized sequence	GGATCCAATCCGAGTAAATCTATTTCGCGTGTGGCACAAGAGTTGTCC<br>AAATATGAAATCTTGAAAAAACTGGATGAAAGTGACACAGAGTCTTA<br>TAGTAGTGTGTATTTATGCAAGAAGAAGGGCGAACACAAACGGTTTG<br>TGTGTAAAATCGTTAAACCGTCCACCTTCAACTCGCTAGAGTTTGACG<br>TGCACATACTGATGCGCAACAACCCCAACTTTATTAAACTGCACAATT<br>TCGTGTTCAACGACAATGGCGAGAGTCTACTCATCATGGACTACGTGA<br>GCGATGGTGATCTCTTTGATTTTGTGAAGATGAACGACACGCGCGAGT<br>TGCGCCTCAACGAAGCCGCATGCAAAAAAATAATCATCACATTGGTGA<br>CGGCGCTCAACGATCTACACAAAAACAATATTGTGCACAACGACGTCA<br>AACTGGAAAACTTGTTGTACGACCGCAAAAAGAAACGACTGTTTGTAT<br>GCGACTACGGACTGTCGAGAATCGTGGGCACACCCTCCTTCTACGACG<br>GCACCACAGTCTACTTTTCACCGGAGAAGATCCGTCATGAAGCGTACC<br>AGACATCGTTCGACTGGTGGGCGGTGGGAGTGGTGGCGTATGAAATCT<br>TATCGACCGAGTACCCGTTTGACATCAACGAGGACAACGAGGAGGAGA<br>TGGACGCCATTGAACCCAAAGACATGTTACCCCTCTACTCTAAACCGCT<br>ACCCACCATTGAACACGTGTCCAAAAAGGCCAACGATTTTGTTAGGCGC<br>ATGCTGGCACTAGACATCAATAGTAGACTGAGCACCTACGATGAAATAA<br>TTAAACACCCGTTCTTATGTTTCTGAATTC

‡ Primer sequence mismatches are shown in upper case and restriction sites underlined

Supplementary Table 2 | X-ray crystallography data collection, refinement and validation statistics

**Data collection parameters**	**PK-1** (PDB-6VVG)
Beamline	MX2 (Australian Synchrotron)
Detector	ADSC Quantum 315r CCD
X-ray wavelength (Å)	0.9537
Space group	$P\ 2_1\ 2_1\ 2_1$
Unit cell *a*, *b*, *c* (Å)	41.94, 115.37, 119.39
Unit cell α, β, γ (°)	90, 90, 90
Resolution range (Å)	39.57–2.01 (2.08–2.01)‡
No. of total reflections	486,615 (26,454)
No. of unique reflections	38,366 (3154)
Multiplicity	12.7 (8.3)
Completeness (%)	97.1 (81.4)
R_{merge}	0.28 (1.29)
$CC_{1/2}$ (%)	99.4 (54.2)
I/σ (I)	10.0 (1.6)
**Refinement statistics**	
No. of reflections used	38,366 (3152)
No. of reflection used in test set	1984 (161)
R_{work}/R_{free} (%)	19.29/22.48
Total non-H atoms	4,777
Protein	4,307
AMP	46
Water	424
Mean *B* factor (Å^2)	
Protein	23.3
AMP	58.9
Water	27.9
r.m.s deviations	
Bond (Å)	0.007
Angle (°)	0.79
***MolProbity* statistics†**	
Rotamer outliers (%)	0
Clashscore	2.90
Ramachandran plot	
Favored/allowed regions (%)	98.07/1.93

‡ Values in parentheses are for the highest resolution shell

† *MolProbity* structure-validation web service[5]

Supplementary Table 3 | AUC data collection and analysis statistics

**Sedimentation velocity analysis**	
Sedimentation coefficient (S)	4.13
Sedimentation coefficient standardized to 20° in water ($S_{20,w}$)	4.40
Frictional ratio (f/f_0)	1.29
Measured molar mass (kDa)	66.87
Calculated dimer molar mass from sequence (kDa)	66.04
Fit r.m.s.d.	0.0014
Partial specific volume (mL g^{-1})	0.7407
Buffer density (g cm^3)	1.00800
Buffer viscosity (cP)	1.0355

Supplementary Table 4 | SAXS data collection and analysis statistics

**Data collection parameters**	
Instrument	Australian Synchrotron SAXS/WAXS beamline
Detector	PILATUS3-2M (Dectris)
Detector distance (mm)	3
Wavelength (Å)	1.0332
Total q range (Å^{-1})	0.005 – 0.5
Maximum flux at sample	8 x 10^{12} photons per second at 12 keV
Exposure time	Continuous 1 second frame measurements
Sample configuration	SEC-SAXS with co-flow
Temperature	12
**Analysis statistics**	
$I(0)$ (cm^{-1}) (from Guinier analysis)	0.014 ± 2.7e-05
R_g (Å) (from Guinier analysis)	30.27 ± 0.23
R_g (Å) (from $P(r)$ analysis)	30.15 ± 0.15
D_{max} (Å)	96
Porod volume estimate (Å^3)	94,544
**Molecular mass (MM) determination**	
MM (from Porod Volume, kDa)	55.6
MM (from SAXSMoW2*, $q_m = 8/R_g$, kDa)	74.3
Calculated dimer MM from sequence (kDa)	66.0
**Software employed**	
Primary data reduction	ScatterBrain (Australian Synchrotron)
Data processing	PRIMUSQT (ATSAS)
Computation of model intensities	CRYSOL

*http://saxs.ifsc.usp.br/

Supplementary Table 4 | *In vitro* kinase assay in numerical format

PK-1 construct	Luminescence intensity				
	Replicate 1	Replicate 2	Replicate 3	Mean	SD
Wild type	47621	52626	54162	52626	3420
PK-1$^{15\text{-}279}$ (Monomer)	15394	11897	9136	11897	3136
PK-1$^{15\text{-}279}$ (Dimer)	37265	30301	38010	37265	4252
K49A	24779	27390	22425	24779	2484
D141N	3461	3327	3884	3461	291
D160N	5535	5429	4581	5429	523
E^{217}DNEEE>Q^{217}NNQQQ	57013	55140	51329	55140	2897

SUPPLEMENTARY REFERENCES

1. Young MA*, et al.* Structure of the kinase domain of an imatinib-resistant Abl mutant in complex with the Aurora kinase inhibitor VX-680. *Cancer Res* **66**, 1007-1014 (2006).

2. Cowan-Jacob SW*, et al.* Structural biology contributions to the discovery of drugs to treat chronic myelogenous leukaemia. *Acta Crystallogr D Biol Crystallogr* **63**, 80-93 (2007).

3. Neuwald AF. The CHAIN program: forging evolutionary links to underlying mechanisms. *Trends Biochem Sci* **32**, 487-493 (2007).

4. Neuwald AF. A Bayesian sampler for optimization of protein domain hierarchies. *J Comput Biol* **21**, 269-286 (2014).

5. Chen VB*, et al.* MolProbity: all-atom structure validation for macromolecular crystallography. *Acta Crystallogr D* **66**, 12-21 (2010).
