# Supplementary material for: Granulovirus PK-1 kinase activity relies on a side-to-side dimerization mode centered on the regulatory αC helix
Source: Nat Commun. 2021 Feb 12;12:1002. doi: 10.1038/s41467-021-21191-7 (PMC7881018; doi:10.1038/s41467-021-21191-7)
Supplement: Supplementary file 3 — Description of Additional Supplementary Files [file 41467_2021_21191_MOESM3_ESM.pdf]

Description of Additional Supplementary Files

File Name: Supplementary Movie 1
Description: Molecular dynamics simulation of *Cp*GV PK-1 dimer
Accompaniment to Fig. 3d-e.

File Name: Supplementary Movie 2
Description: Molecular dynamics simulation of *Cp*GV PK-1 monomer
Accompaniment to Fig. 3d-e.
